# Behavioral interventions to reduce particulate matter exposure in patients with COPD

**DOI:** 10.1097/MD.0000000000028119

**Published:** 2021-12-10

**Authors:** Jieun Kang, Ji Ye Jung, Jin-Young Huh, Hyun Woo Ji, Hwan-Cheol Kim, Sei Won Lee

**Affiliations:** a Division of Pulmonary and Critical Care Medicine, Department of Internal Medicine, Ilsan Paik Hospital, Inje University College of Medicine, Goyang, Republic of Korea; b Division of Pulmonary and Critical Care Medicine, Department of Internal Medicine, Severance Hospital, Yonsei University College of Medicine, Seoul, Republic of Korea; c Department of Pulmonary and Critical Care Medicine, Asan Medical Center, University of Ulsan College of Medicine, Seoul, Republic of Korea; d Department of Occupational and Environmental Medicine, Inha University Hospital, Inha University College of Medicine, Incheon, Republic of Korea.

**Keywords:** chronic obstructive pulmonary disease, intervention, particulate matter

## Abstract

**Introduction:**

Chronic obstructive pulmonary disease (COPD) is commonly affected by particulate matter (PM) exposure. In this study, we aimed to evaluate whether behavioral interventions to reduce PM exposure improve clinical outcomes in patients with COPD.

**Methods:**

A multicenter randomized controlled trial will be conducted involving 120 participants recruited from 3 hospitals in the capital region of the Republic of Korea. Patients aged 40 to 80 years with a diagnosis of COPD and a forced expiratory volume at 1 s <80% of the predicted value are eligible for inclusion. The participants will be randomized to either the intervention group or the usual care group (2:1). The behavioral interventions will comprise the following activities: checking air quality forecast; operating indoor air cleaners and regular check-ups of filters; ventilating the home regularly by opening windows; adhering to inhaler treatment; and refraining from going out on high air pollution days. “Internet-of-things”-based, gravimetric, and light-scattering methods will be used to measure indoor and outdoor PM concentrations. To estimate the degree of individual PM exposure, a time-activity diary and land use regression modeling will be used. The efficacy of the behavioral interventions on the following outcomes will be analyzed: amount of PM exposure, changes in forced expiratory volume at 1 s from the baseline, changes in respiratory symptoms and quality of life, risks of exacerbation, hospitalization, and death.

**Discussion:**

Given the harmful effect of air pollutants, individual-level interventions to reduce exposure may be significant. However, there is a lack of evidence on how effective such interventions are to date. This study will be able to provide physicians and patients with evidence-based strategies to reduce PM exposure in daily life.

**Trial registration number:**

NCT04878367

## 1 Introduction

Air pollution is undoubtedly a global public health issue with many facets. The World Health Organization estimated in 2016 that air pollution was responsible for 4.2 million premature deaths per year.^[1]^ Among various air pollutants, particulate matter (PM) has been recognized as a significant threat to public and individual health.^[2]^ PM is often classified by size fractions; PM_10_ and PM_2.5_ refer to particles with aerodynamic diameters of ≤10 μm and ≤2.5 μm, respectively. PM affects patients with chronic respiratory diseases by its penetrability into peripheral lung regions. Chronic obstructive pulmonary disease (COPD), the third leading cause of death worldwide,^[3,4]^ is one of the diseases that are commonly affected by PM exposure.^[5–8]^ According to a previous study, chronic exposure to ambient PM was associated with not only COPD morbidity but also with lower lung function.^[9]^ Ambient PM exposure was also linked to increased mortality,^[7,10]^ hospitalizations,^[6]^ and exacerbations,^[6,11]^ in patients with COPD.

Exposure to PM occurs not only outdoors but also in the indoor environment. Indoor air pollution has a wide range of impact on respiratory health; in particular, the development of COPD has been associated with air pollutants from indoor sources, such as solid fuel combustion and cooking.^[3,12,13]^ PM exposure in the indoor environment also affects the outcomes of COPD; Hansel et al^[14]^ have found that indoor PM_2.5_ concentrations in homes of former smokers with COPD were associated with an increased risk of severe exacerbations, increased burden of respiratory symptoms, and more frequent rescue medication use. As individuals spend majority of their time indoors, the health effects of PM should be evaluated considering air pollutant exposure not only outdoors but also in indoor environments.

Although hazardous outcomes of PM are recognized, evidence-based strategies for patient guidance are not concrete. There is a lack of research to date to support whether personal-level behavioral interventions to reduce PM exposure can improve clinical outcomes of patients with COPD. In a pilot study, the relationship between various daily behaviors aimed to reduce PM exposure and indoor/outdoor PM_2.5_ concentrations was investigated.^[15]^ The study found that a few behaviors, including checking air quality forecast and using an air cleaner, were associated with reduced indoor PM_2.5_ concentrations compared to outdoor concentrations. Based on the findings, we aim to evaluate whether the behavioral interventions and performances of certain protective measures in daily life improve clinical outcomes in patients with COPD.

## 2 Methods

### 2.1 Study design

**Figure 1:**
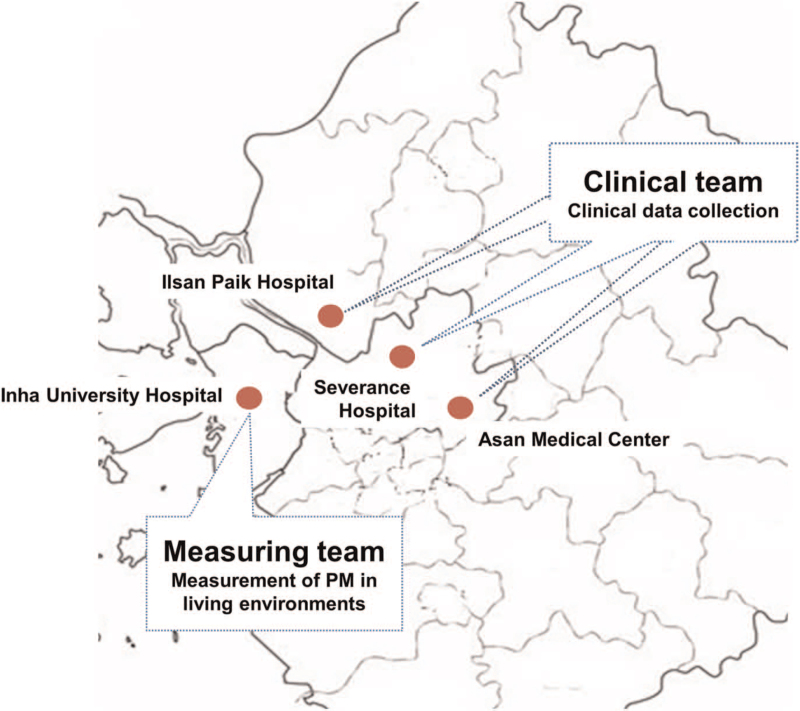
Study sites. Participants will be enrolled from 3 academic institutions located in the capital region of the Republic of Korea: Asan Medical Center (Seoul), Severance hospital (Seoul), and Ilsan Paik Hospital (Goyang). PM measuring devices will be installed and analyzed by the measuring team from Inha University Hospital. PM = particulate matter.

This study is a multicenter randomized controlled trial evaluating the efficacy of behavioral interventions to reduce PM exposure in patients with COPD. A total of 120 patients will be enrolled from 3 academic institutions located in the capital region of the Republic of Korea (Fig. 1): 60 from Asan Medical Center (Seoul), 30 from Severance hospital (Seoul), and 30 from Ilsan Paik Hospital (Goyang). The study duration is 1 year and study participants will be followed up on every 3 months after a screening visit. This study was approved by the Institutional Review Board of each site: Asan Medical Center (2021-0701), Severance Hospital (4-2021-0607), and Ilsan Paik Hospital (2021-05-042). All of the participants will be asked to provide their written informed consent after receiving comprehensive information about the study. Additional informed consent will be obtained for the collection and use of biological specimens.

### 2.2 Study participants

Patients who visit the outpatient clinics at each site will be screened for enrolment. The inclusion criteria of the study will be as follows: patients aged ≥40 years; those with a COPD diagnosis (postbronchodilator forced expiratory volume at 1 s [FEV_1_]/forced vital capacity <0.7); and those with FEV_1_ <80% of the predicted value. The following patients will be excluded: aged ≥80 years; have no respiratory symptoms; are unable to respond to questionnaires; or cannot understand air sampler device instructions.

### 2.3 Randomization and intervention

**Figure 2:**
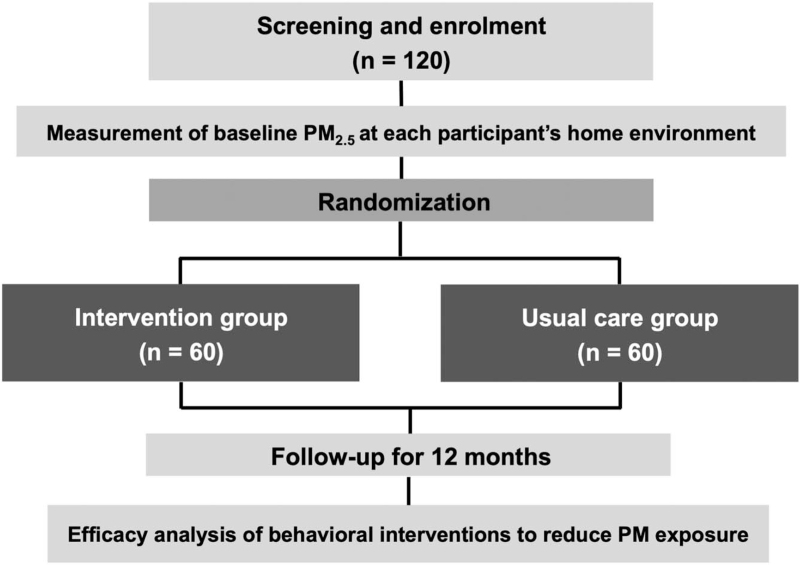
Study flow. PM = particulate matter.

The study flow is shown in Figure 2. After enrolment, indoor PM_2.5_ concentrations will be measured for 1 month for each participant (baseline indoor PM_2.5_ concentrations). Study participants will be randomly allocated (1:1) either to the intervention group or the usual care group using the baseline monthly PM_2.5_ concentrations and baseline FEV_1_. The behavioral interventions will comprise the following 5 activities: checking the air quality forecast; operating an indoor air cleaner and regular check-up of filters; ventilating their home regularly by opening windows; adhering to inhaler treatment; and refraining from going out on high air pollution days. The intervention group will receive education at each visit on the importance of their behavioral change and will be advised to complete checklists everyday as part of their compliance with the interventions. Air cleaners will be rented to participants in the intervention group during the study period if they do not have one. The usual care group will continue to receive the treatment they were receiving but without any instructions on behavioral changes.

### 2.4 Data collection

The participants’ demographics and clinical data, including age, sex, body mass index, smoking status, medical history (including asthma), and exacerbation history will be obtained at enrolment. The participants will visit their respective hospitals every 3 months for 1 year. At each visit, the participants will perform pre- and postbronchodilator spirometry. Their COPD assessment test, modified Medical Research Council, and St. George Respiratory Questionnaire for patients with COPD scores will be obtained to assess respiratory symptoms and health-related quality of life. Development of acute exacerbation will be checked monthly via a telephone interview and/or at the regular follow-up visit. Any changes in inhaler medications or hospitalizations for respiratory causes will be monitored. Sputum samples will be obtained at each visit and blood samples, at the end of the study. The timeline of data collection is shown in Table 1.

**Table 1:** Timeline of data collection.

	Screening	Visit 1	Visit 2	Visit 3	Visit 4
	0 mo	3 mo	6 mo	9 mo	12 mo
Clinical data					
PFT	+	+	+	+	+
CAT, mMRC	+	+	+	+	+
SGRQ	+	+	+	+	+
Exacerbation	Monthly				
Individual environmental risk assessment					
Residence	+				
Activity		+	+	+	+
Protective behavior	+	+	+	+	+
Indoor PM measurement					
Gravimetric method		+		+	
Light scattering method		+	+	+	+
Portable device		+	+	+	+
IoT-based device	Continuous monitoring				
Outdoor PM measurement					
IoT-based device	Continuous monitoring				
Air pollution network	Continuous monitoring				
Samples					
Sputum		+	+	+	+
Blood					+

#### 2.4.1 Residential environment assessment

Study participants will complete a detailed questionnaire about their residential environment at enrolment. The outdoor environmental assessment will include the following: the location of their current residence; distance from the road; traffic volume; and time spent outside of the house at weekdays and weekends. Collected information on their indoor environment will include the following: type of housing; year of construction; number of family members residing together; number of rooms; methods for heating and cooling; methods of indoor ventilation; presence and types of household appliances (air cleaner, air conditioner, humidifier, dehumidifier, gas heater, oil-filled heater, and fireplace); air cleaner operating time; whether the kitchen and living room are separated; use of ventilating fan when cooking; type of flooring; presence of curtains; presence of sofa and type of material; presence of mold; whether participants have recently moved; any renovation or reconstruction works; presence and type of pet; presence and type of plants; methods of cleaning and frequency; and exposure to indoor secondhand smoking.

#### 2.4.2 Indoor and outdoor PM exposure assessment

“Internet-of-things (IoT)”-based PM measuring devices (CP-16-A5; Aircok Inc., Seoul, Republic of Korea) will be installed inside and outside of the house for year-round monitoring of indoor and outdoor PM concentration levels. Gravimetric and light-scattering methods will also be performed for more precise indoor PM measurement. A mini-volume air sampler (Model KMS-4100; KEMIK Corp., Seongnam, Republic of Korea), MicroPEM (RTI International, Research Triangle Park, NC), and dust spectrometer (11-D; GRIMM Aerosol Technik Ainring GmbH & Co. KG, Ainring, Germany) will be installed at the participants’ homes for 24 hours every 3 months. The devices will be installed in the room the participant reportedly spend the most time in. When these devices are installed, the participants will be asked to record a time-activity diary and carry a portable PM measurement device (Airbeam2; HabitatMap, Inc., Brooklyn, NY) to assess their time spent indoors and outdoors and to estimate individual exposure to PM. Outdoor measurement from the IoT-based device will be complemented with the information from Air Korea, a national air pollution information system in the Republic of Korea (http://www.airkorea.or.kr) that provides data on the PM concentrations relating to the residential address.

#### 2.4.3 Statistical estimation for unmeasured PM exposure

Although outdoor PM concentrations will be measured with the aforementioned devices, it will be necessary to estimate individual exposures not identified during the actual measurement period. For this, land use regression (LUR) modeling, a popular method for exposure estimation, will be applied.^[16–18]^ LUR models incorporate monitoring data and land-use variables, such as road type, traffic volume, population density, altitude, and topography data obtained using Geographic Information Systems. The LUR modeling that will be used for this study was described in a previous study.^[19]^ The microenvironmental modeling (Stochastic Human Exposure and Dose Simulation) will be applied to estimate distributions of microenvironmental PM concentrations and exposures for participants (Fig. 4).^[20]^

### 2.5 Study outcomes

**Figure 3:**
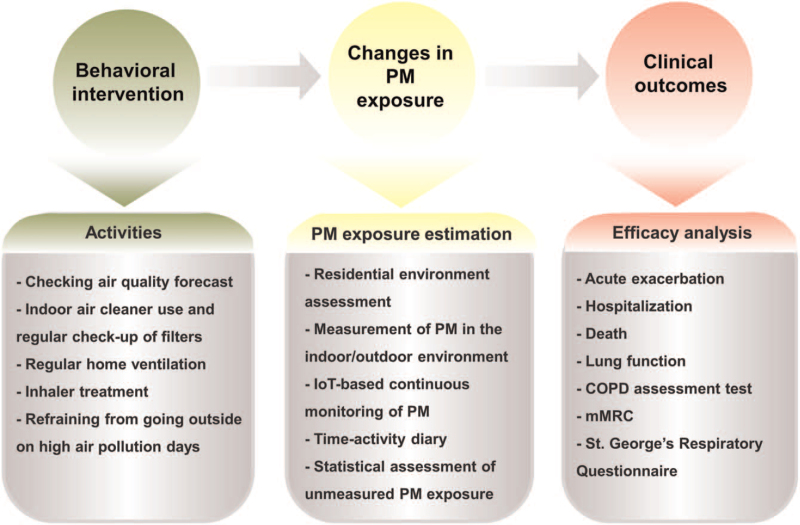
Study concept. This study aims to evaluate the efficacy of behavioral interventions to reduce PM exposure and its effect on the clinical outcomes of patients with COPD. We will examine if the behavioral interventions are associated with a reduction in individual PM exposure and ultimately with improved clinical outcomes. COPD = chronic obstructive pulmonary disease, IoT = internet-of-things, mMRC = modified Medical Research Council, PM = particulate matter.

We will investigate if the behavioral interventions reduce the degree of PM exposure and result in improved clinical outcomes compared with the usual care. The study outcomes are risks of acute exacerbation, respiratory-related hospitalization, death, difference in FEV_1_ from the baseline, and changes in the COPD assessment test, modified Medical Research Council, and St. George Respiratory Questionnaire for patients with COPD scores. The study concept is illustrated in Figure 3.

**Figure 4:**
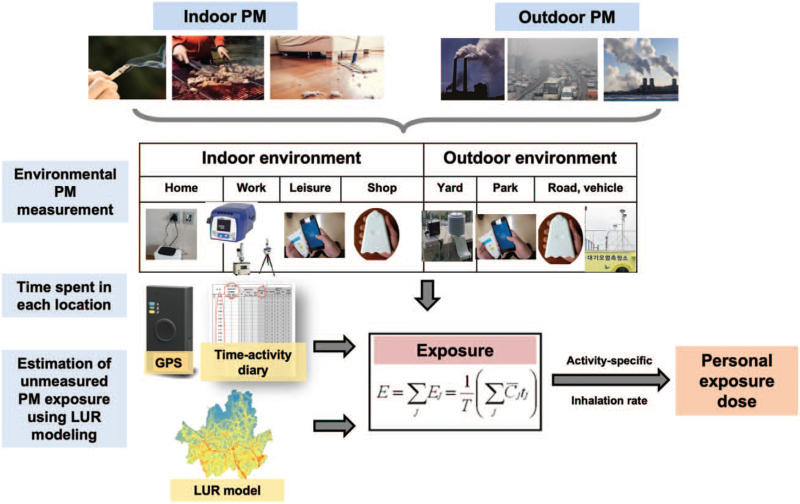
Individual PM exposure estimation. The individual PM exposure estimation will be performed based on the concept of the Stochastic Human Exposure and Dose Simulation model. GPS = global positioning system, LUR = land use regression, PM = particulate matter.

### 2.6 Statistical analysis

Data will be analyzed using SAS software (SAS Institute Inc., Cary, NC) and R software (version 3.5.2; R Development Core Team, Vienna, Austria). Data will be presented as means ± standard deviation or median [interquartile range] for continuous variables and percentages for categorical variables. The Student *t* test or Mann–Whitney test will be used for continuous variables, and the chi-squared test and Fisher exact test will used to compare categorical variables. *P*-values of <.05 will be considered of statistical significance. The risk of acute exacerbation and the incidence of respiratory hospitalization will be compared in the intervention group and the non-intervention group (Chi-squared test or Fisher exact test).

## 3 Discussion

While public policy has a primary role in reducing air pollution, personal lifestyle modification may reduce the harmful effects of PM. This study aims to evaluate whether behavioral interventions can contribute to a reduction in PM exposure and result in improved clinical outcomes in patients with COPD. Through prospectively collected data, including PM concentrations in the indoor and outdoor environments, pulmonary function test results, and patients’ reported outcomes, this study will be able to provide physicians and patients with evidence-based strategies to reduce PM exposure in daily life.

One of the popular interventions that has been studied so far to reduce PM exposure is air cleaner use. A short-term use of air cleaners in college dormitories was associated with a decrease in circulating inflammatory and thrombogenic biomarkers in 35 healthy young adults in China.^[21]^ In another study, the use of air cleaners in classrooms resulted in a significant reduction in PM_2.5_ concentrations and a modest improvement in peak flow in children with asthma, although there were no significant changes in FEV_1_ and asthma symptoms.^[22]^ Air cleaner use was also associated with cardiopulmonary benefits in a crossover study conducted in Canada; there was an increase in FEV_1_ and a decrease in systolic and diastolic pressures.^[23]^ However, there is a lack of research to date on actual health outcomes of air cleaner use in sensitive individuals, such as those with COPD. Very recently, the use of air cleaners with high efficiency particulate air and carbon filters has been shown to significantly reduce indoor PM_2.5_ concentrations and decrease the risk of moderate exacerbations and respiratory symptoms^[24]^; these suggest potential benefits of lifestyle interventions and highlight the importance of further relevant studies in patients with COPD.

Appropriate personal protective equipment may be a useful way of avoiding adverse effects of ambient air pollutants.^[25,26]^ In a study conducted in India, after wearing a facemask for 30 d, peak expiratory flow rate was found to be almost 10% higher than that of the baseline in two-wheeler riders.^[27]^ Wearing a facemask on a day with high air pollution is a protective measure that has been particularly emphasized in East Asia.^[28,29]^ However, the intervention studies of facemasks involved mostly healthy volunteers, and there is no recommended guidance to date for facemasks in preventing the hazardous effects of air pollution.^[30]^ Due to the lack of evidence, the use of facemasks may be limited by individual and public acceptability depending on the cultural difference or perception of discomfort.

Given the harmful effect of air pollutants, individual-level interventions to reduce exposure seem reasonable. However, there is a lack of evidence on how effective such interventions are.^[31]^ When assessing the efficacy of interventions, it should be considered that they may have unintended negative consequences; for example, refraining from going outside to avoid exposure to air pollution may lead to diminished physical activity. Staying indoors may not be helpful if the indoor air pollution level is high. Therefore, multiple/combined risk reduction behaviors against both outdoor and indoor PM exposure should be considered. We selected multiple behaviors based on a previous study result.^[15]^

The degree of PM exposure depends not only on the air quality of the indoor/outdoor environments, but also on the percentage of time an individual spends indoors and outdoors. Considering this, we will measure PM levels both inside and outside the house and estimate how much exposure occurs in each environment based on the time-activity diaries that the participants will be asked to record and on the portable measuring device that they will carry. In addition, IoT censor-based monitoring that will be conducted year-round will enable us to analyze the long-term health impacts of PM exposure in patients with COPD. By simultaneously performing gravimetric and light-scattering methods, the accuracy of the measurement will be improved.

In conclusion, this study is a multicenter randomized controlled study that will evaluate the efficacy of behavioral interventions in reducing the effect of PM exposure on the health outcomes of patients with COPD. Indoor PM concentrations, decline in lung function, changes in respiratory symptoms and quality of life, and risks of exacerbation, hospitalization, and death will be compared between the intervention group and usual care group. Given the lack of evidence to support personal-level interventions, this study will provide a scientific basis on how to prevent PM exposure in daily life in patients with COPD.

## Author contributions

**Conceptualization:** Sei Won Lee.

**Data curation:** Hwan-Cheol Kim.

**Formal analysis:** Jieun Kang, Ji Ye Jung, Hwan-Cheol Kim, Sei Won Lee.

**Funding acquisition:** Sei Won Lee.

**Investigation:** Jieun Kang, Ji Ye Jung, Jin-Young Huh, Hyun Woo Ji.

**Writing – original draft:** Jieun Kang, Sei Won Lee.

**Writing – review & editing:** Jieun Kang, Ji Ye Jung, Jin-Young Huh, Hyun Woo Ji, Hwan-Cheol Kim, Sei Won Lee.

## References

[1] World Health Organization. Available at: https://www.who.int/en/news-room/fact-sheets/detail/ambient-(outdoor)-air-quality-and-health. Accessed August 24, 2021.

[2] Manisalidis I Stavropoulou E Stavropoulos A. Environmental and health impacts of air pollution: a review. Front Public Health 2020;8:14. 32154200 10.3389/fpubh.2020.00014 PMC7044178

[3] Global Initiative for Chronic Obstructive Pulmonary Disease. Global strategy for prevention, diagnosis and management of COPD 2021. Available at: https://goldcopd.org/2021-gold-reports/. Accessed August 21, 2021.

[4] World Health Organization. Available at: https://www.who.int/news-room/fact-sheets/detail/chronic-obstructive-pulmonary-disease-(copd)#:∼:text=Key%20facts,%2Dincome%20countries%20(LMIC). Accessed August 24, 2021.

[5] Li MH Fan LC Mao B. Short-term exposure to ambient fine particulate matter increases hospitalizations and mortality in COPD: a systematic review and meta-analysis. Chest 2016;149:447–58. 26111257 10.1378/chest.15-0513

[6] Zhu R Chen Y Wu S. The relationship between particulate matter (PM10) and hospitalizations and mortality of chronic obstructive pulmonary disease: a meta-analysis. COPD 2013;10:307–15. 23323929 10.3109/15412555.2012.744962

[7] Zanobetti A Bind MA Schwartz J. Particulate air pollution and survival in a COPD cohort. Environ Health 2008;7:48. 18847462 10.1186/1476-069X-7-48 PMC2572050

[8] Han C Oh J Lim YH. Long-term exposure to fine particulate matter and development of chronic obstructive pulmonary disease in the elderly. Environ Int 2020;143:105895. 32615346 10.1016/j.envint.2020.105895

[9] Liu S Zhou Y Liu S. Association between exposure to ambient particulate matter and chronic obstructive pulmonary disease: results from a cross-sectional study in China. Thorax 2017;72:788–95. 27941160 10.1136/thoraxjnl-2016-208910 PMC5738534

[10] Moolgavkar SH. Air pollution and daily mortality in three U.S. counties. Environ Health Perspect 2000;108:777–84. 10964799 10.1289/ehp.00108777 PMC1638292

[11] Choi J Oh JY Lee YS. Harmful impact of air pollution on severe acute exacerbation of chronic obstructive pulmonary disease: particulate matter is hazardous. Int J Chron Obstruct Pulmon Dis 2018;13:1053–9. 29681728 10.2147/COPD.S156617 PMC5881527

[12] Eisner MD Anthonisen N Coultas D. An official American Thoracic Society public policy statement: Novel risk factors and the global burden of chronic obstructive pulmonary disease. Am J Respir Crit Care Med 2010;182:693–718. 20802169 10.1164/rccm.200811-1757ST

[13] Kurmi OP Semple S Simkhada P. COPD and chronic bronchitis risk of indoor air pollution from solid fuel: a systematic review and meta-analysis. Thorax 2010;65:221–8. 20335290 10.1136/thx.2009.124644

[14] Hansel NN McCormack MC Belli AJ. In-home air pollution is linked to respiratory morbidity in former smokers with chronic obstructive pulmonary disease. Am J Respir Crit Care Med 2013;187:1085–90. 23525930 10.1164/rccm.201211-1987OC PMC3734614

[15] Kim H Na G Park S. The impact of life behavior and environment on particulate matter in chronic obstructive pulmonary disease. Environ Res 2021;198:111265. 33939981 10.1016/j.envres.2021.111265

[16] Ryan PH LeMasters GK. A review of land-use regression models for characterizing intraurban air pollution exposure. Inhal Toxicol 2007;19: (Suppl 1): 127–33. 17886060 10.1080/08958370701495998 PMC2233947

[17] Jin L Berman JD Warren JL. A land use regression model of nitrogen dioxide and fine particulate matter in a complex urban core in Lanzhou, China. Environ Res 2019;177:108597. 31401375 10.1016/j.envres.2019.108597

[18] Moore DK Jerrett M Mack WJ. A land use regression model for predicting ambient fine particulate matter across Los Angeles, CA. J Environ Monit 2007;9:246–52. 17344950 10.1039/b615795e

[19] Lamichhane DK Leem JH Kim HC. Associations between ambient particulate matter and nitrogen dioxide and chronic obstructive pulmonary diseases in adults and effect modification by demographic and lifestyle factors. Int J Environ Res Public Health 2018;15: doi: 10.3390/ijerph15020363. 10.3390/ijerph15020363 PMC5858432 29463050

[20] Burke JM Zufall MJ Ozkaynak H. A population exposure model for particulate matter: case study results for PM(2.5) in Philadelphia, PA. J Expo Anal Environ Epidemiol 2001;11:470–89. 11791164 10.1038/sj.jea.7500188

[21] Chen R Zhao A Chen H. Cardiopulmonary benefits of reducing indoor particles of outdoor origin: a randomized, double-blind crossover trial of air purifiers. J Am Coll Cardiol 2015;65:2279–87. 26022815 10.1016/j.jacc.2015.03.553 PMC5360574

[22] Jhun I Gaffin JM Coull BA. School environmental intervention to reduce particulate pollutant exposures for children with asthma. J Allergy Clin Immunol Pract 2017;5:154–9. 27641483 10.1016/j.jaip.2016.07.018 PMC5222771

[23] Weichenthal S Mallach G Kulka R. A randomized double-blind crossover study of indoor air filtration and acute changes in cardiorespiratory health in a First Nations community. Indoor Air 2013;23:175–84. 23210563 10.1111/ina.12019

[24] Hansel NN Putcha N Woo H. Randomized clinical trial of air cleaners to improve indoor air quality and COPD health: Results of the CLEAN AIR STUDY. Am J Respir Crit Care Med 2021;doi: 10.1164/rccm.202103-0604OC. 10.1164/rccm.202103-0604OC PMC8886948 34449285

[25] Zhao P Yu KP Lin CC. Risk assessment of inhalation exposure to polycyclic aromatic hydrocarbons in Taiwanese workers at night markets. Int Arch Occup Environ Health 2011;84:231–7. 20506023 10.1007/s00420-010-0551-1

[26] Langrish JP Mills NL Chan JK. Beneficial cardiovascular effects of reducing exposure to particulate air pollution with a simple facemask. Part Fibre Toxicol 2009;6:08. 10.1186/1743-8977-6-8 PMC2662779 19284642

[27] Singh MP Singh VK Patel DK. Face mask application as a tool to diminish the particulate matter mediated heavy metal exposure among citizens of Lucknow, India. Sci Total Environ 2010;408:5723–8. 20855105 10.1016/j.scitotenv.2010.08.041

[28] van Dorn A. Clearing the air: do facemasks protect health? Lancet Respir Med 2017;5:555–6. 28664860 10.1016/S2213-2600(17)30229-1

[29] Rajper SA Ullah S Li Z. Exposure to air pollution and self-reported effects on Chinese students: a case study of 13 megacities. PLoS One 2018;13:e0194364. 29547657 10.1371/journal.pone.0194364 PMC5856349

[30] Jiang XQ Mei XD Feng D. Air pollution and chronic airway diseases: what should people know and do? J Thorac Dis 2016;8:E31–40. 26904251 10.3978/j.issn.2072-1439.2015.11.50 PMC4740163

[31] Laumbach R Meng Q Kipen H. What can individuals do to reduce personal health risks from air pollution? J Thorac Dis 2015;7:96–107. 25694820 10.3978/j.issn.2072-1439.2014.12.21 PMC4311076

